# Infant neurobehavioural consequences of prenatal cigarette exposure: A systematic review and meta‐analysis

**DOI:** 10.1111/apa.15132

**Published:** 2020-01-24

**Authors:** Suzanne Froggatt, Judith Covey, Nadja Reissland

**Affiliations:** ^1^ Department of Psychology Durham University Durham UK

**Keywords:** meta‐analysis, neurobehaviour, prenatal cigarette exposure, systematic review

## Abstract

**Aim:**

Prenatal exposure to cigarettes leads to alterations in brain development during pregnancy. This has an impact on postnatal psychological and behavioural processes, affecting an infant's neurobehavioural profile with little known about which aspects are affected. The evidence was synthesised to assess the effects of prenatal cigarette smoke exposure on neurobehavioural outcomes within the first year of life.

**Methods:**

Six databases were searched (Web of Science Core Collections, MEDLINE, PsycINFO, CINAHL, EBSCOhost eBook Collection and OpenGrey) in November 2018. Eligible studies (n = 17) had to include a measure of prenatal cigarette exposure and a neurobehavioural assessment ≤1 year of age.

**Results:**

In the first year of life, specific areas of neurobehavioural functioning are related to prenatal cigarette exposure with eight out of 10 areas of neurobehaviour having significant medium (negative affect, attention, excitability, irritability and orientation) or small (muscle tone, regulation and difficult temperament) pooled effect sizes. Only lethargy and stress did not show any significant pooled effects.

**Conclusion:**

Prenatal cigarette exposure affects a significant range of behaviours during the first year of life.

**Key notes**

- Neurobehavioural functioning is affected by prenatal cigarette exposure.
- Five areas of neurobehaviour demonstrated significant medium combined effects and three demonstrated significant small combined effects.
- Lethargy and stress did not demonstrate significant combined effects.

## 1 INTRODUCTION

Prenatal exposure to cigarette smoke has lasting postnatal effects including significant increased risk of cognitive impairment and learning difficulties.^1^, ^2^, ^3^ Research suggests two specific toxins in cigarettes are causing these effects, namely carbon monoxide and nicotine. Carbon monoxide crosses the placenta binding to haemoglobin leading to a reduction in blood flow, ultimately impacting brain development and growth.^4^ Similarly, nicotine readily crosses the syncytium, a thin layer of tissue separating maternal and foetal blood.^5^ Although the foetal brain is protected from a range of neurotoxins, it is specifically sensitive to nicotine which targets specific neurotransmitters, leading to cell abnormalities and impaired foetal brain development by affecting synaptic activity.^5^ Since nicotine affects brain development, it has the potential to affect neurobehaviour^6^ including levels of excitability, negative affect, social orientation and regulation in infants.^7^ However, there are a number of potential confounding factors that may influence human infant neurobehaviour, leading to difficulties in underpinning the contribution of cigarettes on the neurobehavioural outcome. Therefore, animal models provide an experimental paradigm to define the mechanisms of nicotine on neurobehaviour. For example, where environmental factors are controlled, rats exposed to nicotine show increased motor activity as well as deficits in cognition, including attentional problems.^1^ From both human and animal research, it appears evident that toxin exposure associated with cigarette smoking leads to alterations in the brain which are reflected in neurobehavioural outcomes.

Neurobehaviour is defined as a bidirectional relationship between biological and behavioural systems, in which behavioural output is moderated by neural feedback.^8^ It is an interaction between biological and psychosocial factors that influence human behaviour.^8^ This definition was originally proposed in order to characterise neurobehaviour in late childhood. However, it also applies to infant assessments of neurobehavioural factors such as the availability and fluctuation of sleep and awake states, muscle tone assessed by items such as pulling the infant to a seated position from lying, irritability and neurological reflexes, for example the Babinski and glabellar responses.^8^, ^9^ Specific measures assessing infant neurobehavioural development include habituation, muscle tone, attention and stress.^10^

Measures of infant behavioural development are often not mentioned in information leaflets on the effects of prenatal tobacco exposure which are distributed to parents; rather, leaflets directed at parents emphasise health opposed to psychological risks of smoking.^11^ Dual emphasis of both the behavioural consequences and health‐related risks associated with smoking is required in order for parents to understand the overall effects of cigarette exposure during pregnancy. Anecdotal experiences of previous healthy uncomplicated pregnancies may lead women to continue smoking during pregnancy.^12^ However, a thorough understanding of neurobehavioural outcomes within the first year of life and the trajectory of later childhood difficulties is essential information that should be provided to parents before and during their pregnancy. Indeed, research indicates that early neurobehavioural functioning may be predictive of later childhood developmental deficits,^13^ particularly for infants who have been exposed prenatally to cigarettes.^14^ There is a growing body of evidence that has assessed the neurobehavioural consequences of prenatal cigarette exposure on infant development during the first year of life.^15^, ^16^ Although reviews have been carried out assessing prenatal exposure on developmental outcomes,^17^, ^18^ the current review is the first meta‐analysis assessing neurobehavioural outcomes within the first year of life. The emphasis is on the first year of life as insults during the critical period of development may have lasting impact, particularly for behaviour and cognition.^19^ During prenatal and early infant development, the brain is rapidly changing in regard to structure and function, with toxins, such as metabolites of cigarettes, altering the programming for healthy behavioural development.^20^ For example, research highlights that scores on a neurobehavioural assessment during infancy had the ability to predict psychomotor development and externalising behaviours at three years of age.^21^ Moreover, by employing meta‐analytic methods to synthesise the results of the existing studies, we can explore which subcategories of neurobehavioural development are most affected.

## 2 METHOD AND MATERIALS

The methodological reporting of this review follows the PRISMA guidelines.

### 2.1 Literature search

In this meta‐analysis, our aim is to identify which subcategories of neurobehaviour are impacted by prenatal cigarette exposure within the first year of life. A literature search of six databases was conducted (Web of Science Core Collections, MEDLINE, PsycINFO, CINAHL, EBSCOhost eBook Collection and OpenGrey) in November 2018. Search terms are listed in Table 1. Although the review focuses on tobacco exposure, nicotine was included as a term to make the search more exhaustive.^22^

**Table 1:** Web of Science Core Collections search strategy

Search terms	
Web of Science Core Collections (*k* = 1190) 1950‐2018	
Initial search	Maternal smoking pregnancy
	Prenatal nicotine exposure
	Prenatal tobacco exposure
	Prenatal cigarette exposure
	Prenatal smoke exposure
	Foetal nicotine exposure
	Foetal tobacco exposure
	Foetal cigarette exposure
Searched within *(separately for each phrase)*	Affect (*k* = 208) Attention (*k* = 130) Behaviour (*k* = 127) Cognition* (*k* = 158) Emotion (*k* = 62) Excitability (*k* = 0) Irritability* (*k* = 4) Lethargy (*k* = 1) Motor* (*k* = 46) Muscle tone (*k* = 7) Neurobehaviour* (*k* = 30) Neurodevelopment* (*k* = 53) Orientation (*k* = 5) Regulation (*k* = 33) Social (*k* = 198) Stress (*k* = 20) Temperament (*k* = 8)
Applicable once duplicates removed: 809	

Note

Published articles are restricted from 1950 to 2018, with unpublished research having no time limits. The language was set to English. No methodological limits were applied.

### 2.2 Study selection

Studies were included if they reported a measure of both prenatal exposure to cigarettes and postnatal neurobehavioural measurements at ≤1‐year post birth. A number of exclusions were in place, including animal studies, reviews (systematic, literature and meta‐analyses), children >1 year of age, studies with no record of maternal prenatal cigarette use, studies focusing on medical, health or birth outcomes and studies using nicotine replacement therapy. The database searches were combined, and duplicate records were removed. The studies were screened by the primary author to assess whether they met the inclusion criteria. Full‐text articles were reviewed for further analysis of study inclusion criteria. The reference lists of these papers were screened for any additional articles. Abstracts and articles were reviewed with the third author.

### 2.3 Data extraction and assessment of methodological quality

A pre‐defined extraction sheet was used to record study characteristics. Extracted information included (a) main outcome measure, (b) participant characteristics (number of infants, infant age, number prenatally exposed and number not exposed), (c) tobacco measurement, (d) controls and (e) results. Where an effect size (Cohen's *d*) was not provided, it was calculated from the available data using the Campbell Collaboration effect size calculator (https://campbellcollaboration.org/effect-size-calculato.html). Where possible effect sizes were based on analyses in which potentially confounding variables such as preterm birth, gestational age at birth, maternal demographics, and substance use (eg alcohol),^23^, ^24^ had been taken into consideration (Table 2). Risk of bias for individual studies was calculated using the ROBINS‐I tool^25^ (Table S1).

**Table 2:** Studies included within the analysis

Reference/country	Number of infants	Infant age	Assessment	Subcategory	Effect size (Cohen's *d*)	Covariates controlled for in the analysis	Overall bias
Barros et al^10^ Brazil	388 infants (365 not exposed, 23 exposed)	24‐72 h old	NICU Network Neurobehavioural Scale	Attention	−1.3	Anaesthesia at birth, type of delivery, gender, age of newborn at assessment, time since last feed and duration of assessment	Low
				Excitability	−0.636		
				Lethargy	−1.142		
				Stress	−0.587		
Espy et al^56^ USA	304 infants (161 not exposed, 143 exposed)	2 d old	Neonatal Temperament Assessment	Attention	−0.465	Mothers' IQ estimate. Marital status, maternal age, education, income, alcohol intake, newborn gender, race, SHS exposure, medication use, gravida, parity, weight gain, maternal health, delivery health, BSI summary index, CAARS:S Attention Deficit/Hyperactivity Disorder index and BIA IQ estimate	Low
				Irritability	−0.192		
Godding et al^57^ Belgium	33 infants (16 not exposed, 17 exposed)	Up to 5 d old	Neurological Scores and Finnegan Withdrawal Scores	Muscle tone	−0.3785	Term of pregnancy and feeding method	Low
Hernandez‐Martinez et al^34^ Spain	265 infants (203 not exposed, 62 exposed)	48‐72 h old	Neonatal Behavioural Assessment Scale	Negative affect	−0.02	Socio‐economic status, birthweight and gestational age. Maternal age, socio‐economic status, newborn gender, birthweight, gestational age, Apgar scores, parity, delivery type, trait anxiety	Low
				Excitability	−0.44		
				Orientation	−0.35		
				Regulation	−0.351		
King et al^58^ USA	48 infants (24 not exposed, 24 exposed)	3‐5 mo	Response to bell ring, brain response	Orientation	−0.8471	Maternal education, gestation at birth, age at assessment, birthweight, ethnicity	Moderate
Law et al^32^ USA	56 infants (29 not exposed, 27 exposed)	Between 36 and 41 wk gestational age	NICU Network Neurobehavioural Scale	Excitability	−0.829	Parity, 5‐min Apgar scores and birthweight. Maternal age, gravida, education, employment, socio‐economic status, alcohol use, gestational age, Apgar score at 1 min	Low
				Muscle tone	−0.711		
				Stress	−1.510		
Mansi et al^31^ Italy	50 infants (25 not exposed, 25 exposed)	56‐72 h old	Neonatal Behavioural Assessment Scale	Attention	−1.358	Gender, gestational age, postnatal age, birthweight, Apgar scores, bilirubin	Low
				Irritability	−1.949		
				Muscle tone	−1.010		
				Orientation	−1.115		
				Regulation	−0.599		
Mundy^39^ UK	71 infants (47 not exposed, 24 exposed)	6 mo	Laboratory Temperament Assessment Battery and Infant Behaviour Questionnaire	Difficult temperament	−0.556	None noted	Moderate
Mundy^39^ UK	71 infants (47 not exposed, 24 exposed)	6 mo	Laboratory Temperament Assessment Battery and Infant Behaviour Questionnaire	Negative affect	−0.409	None noted	
				Difficult temperament	−0.399		
Pickett et al^37^ UK	15 943 infants (11 747 not exposed, 4196 exposed)	9 mo	Carey Infant Temperament Scale	Negative affect	−0.759	None noted	Moderate
				Orientation	−0.070		
				Regulation	−0.114		
				Difficult temperament	−0.134		
Saxton^38^ UK	32 infants (17 not exposed, 15 exposed)	4‐6 d old	Neonatal Behavioural Assessment Scale	Orientation	−0.8471	None noted	Moderate
				Regulation	−0.782		
Schuetze et al^29^ USA	115 infants (46 not exposed, 69 exposed)	2‐4 wk old and again at 7 mo old	Infant Behaviour Questionnaire	Negative affect	−0.806	Mothers' age, education, socio‐economic status, parity, number of prenatal visits, substance use, infant birthweight, head circumference and birth length	Low
Shisler et al^30^ USA	258 infants (77 not exposed, 181 exposed)	2 and 9 mo old	Focused attention assessment and behavioural reactivity	Attention	−0.238	Mothers' age, education, prenatal alcohol and marijuana, partner status, birthweight, gestational age, gender, head circumference at birth	Low
Stroud et al^35^ USA	56 infants (28 not exposed, 28 exposed)	17 d old	NICU Network Neurobehavioural Scale	Excitability	−0.665	Maternal SHS exposure, infant SHS exposure, feeding, maternal depression, socio‐economic status, maternal age and depression	Low
				Regulation	−0.565		
Stroud et al^48^ USA	962 infants (366 not exposed, 596 exposed)	<3 d old	Graham‐Rosenblith Behavioural Examination	Irritability	−0.125	Maternal age, race, socio‐economic status, birthweight and infant age at assessment. Gravida, parity, Apgar score at 1 min and Apgar score at 5 min	Low
				Muscle tone	−0.308		
Wiebe et al^59^ USA	218 infants (91 not exposed, 127 exposed)	6 mo old	A battery of assessments including attention, regulation and inhibition	Orientation	−0.236	Propensity scores—alcohol in first month of pregnancy, maternal age, education, IQ, hyperactivity. Parental stress and infant exposure	Moderate
Yolton et al^22^ USA	251 infants (218 not exposed, 33 exposed)	5 wk old	NICU Network Neurobehavioural Scale	Attention	−0.134	Birthweight, age at assessment and infant gender. Maternal age, income, employment, education, marital status, parity, marijuana and alcohol use, maternal blood lead in pregnancy and weight change since birth and maternal depression	Low
				Lethargy	−0.147		
				Regulation	−0.067		
				Stress	−0.002		

### 2.4 Data analysis

Studies that were eligible for the review were grouped according to 10 different subcategories of outcome measures: negative affect, attention, excitability, irritability, lethargy, muscle tone, orientation, regulation, stress and difficult temperament. To be included in the meta‐analysis, the assessment measures had to be similar across the subcategory. For subcategories of neurobehaviour to be included within the analysis, two or more studies were required.^26^ The fail‐safe N method was used to identify any publication bias by providing an estimate of the number of missing studies that would need to be published with an effect size of *d* = 0 for the pooled effect size to not be significant.^27^

## 3 RESULTS

### 3.1 Selection of studies

**Figure 1:**
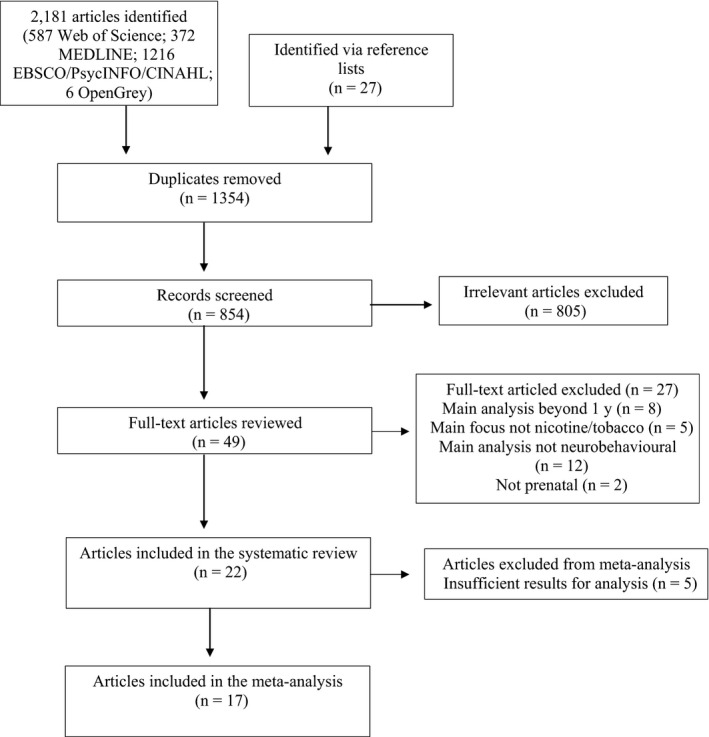
PRISMA flow diagram of studies

The search resulted in 2208 studies. After removal of duplicates, 854 studies were reviewed in terms of title and abstract, resulting in 49 eligible studies which were subjected to a full‐text review. These articles were reviewed in‐depth, checking for a measure of prenatal smoke exposure and a postnatal neurobehavioural measure, and 27 articles were removed leaving 22 articles that based on our selection criteria could be included in the review (see Figure 1). Five of these articles reported insufficient data leaving 17 articles included in the meta‐analysis. Authors of the five studies reporting insufficient results were contacted, where possible, to obtain further details. However, this was unsuccessful. See Figure 1 for flow diagram of study selection and Table 2 for details of the studies included in the analysis.

### 3.2 Study characteristics

The 17 studies included in the meta‐analysis analysed 19 162 infants. There were 5672 infants exposed to cigarettes prenatally and 13 490 who had no prenatal cigarette exposure. Studies came from six different countries: USA (n = 9), UK (n = 4), Spain (n = 1), Italy (n = 1), Brazil (n = 1) and Belgium (n = 1). To assess level of maternal or infant smoke exposure, studies used either a questionnaire method (n = 7), biological measures such as cotinine levels via saliva (n = 2) or a combination of the two methods (n = 8). Nine different assessment scales were used to measure a range of neurobehaviours. Details of the assessments are in Table 3.

**Table 3:** Assessment measures

Assessment measure	Number of studies using assessment	Details
NICU Network Neurobehavioural Scale (NNNS)	4	This assessment was designed to capture the vulnerabilities of high‐risk infants exposed to toxic substances and for newborns between 30 and 46 wk gestational age. Raw data were used to create summary scores based on 13 dimensions including attention, arousal, excitability, hypertonicity, hypotonicity, lethargy, regulation, handling, stress and reflexes^22^
Neonatal Behaviour Assessment Scale (NBAS)	3	Assesses early regulatory behaviour.^56^ State changes are provoked and the infants' habituation, self‐consoling abilities and reflexes. It includes 28 behavioural items and 18 reflexes. Items given a score include motor abilities, habituation, orientation, reflexes and regulation^31^
Carey Infant Temperament Scale	1	The scale assesses three areas of temperament: positive mood, receptivity to novelty and regularity^37^
Infant Behaviour Questionnaire‐Revised	2	This is a parental report questionnaire for infants between 3 and 12 mo of age. There are four main subcategories of this scale including extroversion, negative affect, orientation and regulation^28^
Graham‐Rosenblith Behavioural Examination	1	This is a standardised assessment which involves observation and manipulation of the infant to assess reflexes, muscle tone and responses to stimulation. Additionally, measures of irritability and signs of neurological damage are assessed^35^
Laboratory Assessment Battery (Lab‐TAB)	2	Designed to assess early infant temperament^39^
Finnegan Withdrawal Scale	1	Evaluation of the central nervous system function and respiratory functions^57^
Neurological Scores	1	Assesses a range of abilities including muscle tone, reflexes, for example sucking, stepping reactions and alertness, for example eye opening^57^
Neonatal Temperament Assessment (NTA)	1	The assessment assesses early regulatory behaviours^56^

### 3.3 Neurobehavioural subcategory analysis

**Figure 2:**
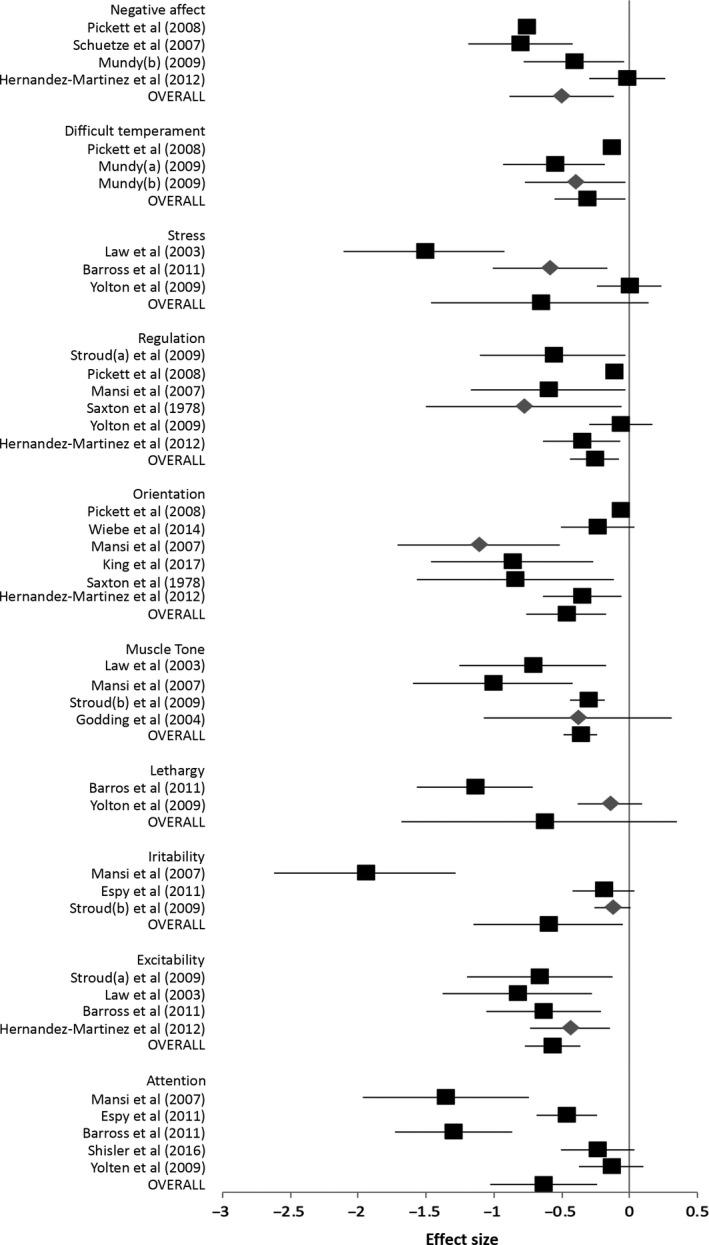
Forest plot of analysis

See Figure 2 for forest plot of results and Table 4 for subcategory analysis.

**Table 4:** Subcategory analysis

Subcategory	Number of studies	Assessment measures	Cohen's *d*	95% CI	*Z*	*P* value (*Z*)	*Q*	*P* value (*Q*)
Negative affect*	4	NBAS, Lab‐TAB, Carey Infant Temperament Scale, Infant Behaviour Questionnaire‐Revised	−0.5027	−0.8863, −0.1191	−2.5685	.0102	28.2227	<.001
Attention*	5	NBAS, NICU Network Neurobehavioural Scale, NTA	−0.6352	1.0318, −0.2386	−3.1292	.001	32.4514	<.001
Excitability*	4	NICU Network Neurobehavioural Scale, NBAS	−0.5697	−0.7726, −0.3678	−5.5296	<.001	1.8737	.599
Irritability*	3	NICU Network Neurobehavioural Scale, Graham‐Rosenblith Behavioural Examination, NTA	−0.6003	−1.1486, −0.0519	−2.1456	.0319	27.185	<.001
Lethargy	2	NICU Network Neurobehavioural Scale	−0.6280	−1.680, 0.3469	−1.2625	.2068	15.8478	.001
Muscle tone*	4	NICU Network Neurobehavioural Scale, Graham‐Rosenblith Behavioural Examination, NBAS, Neurological Scores	−0.3619	−0.4842, −0.2395	−5.7964	<.001	6.9088	.0749
Orientation*	6	NBAS, Carey Infant Temperament Scale	−0.4645	‐0.7577, 0.1713	−3.1047	.001	26.9692	.009
Regulation*	6	NICU Network Neurobehavioural Scale, NBAS, Carey Infant Temperament Scale	−0.2619	−0.4411, −0.0827	−2.864	.004	11.2507	.0465
Stress	3	NICU Network Neurobehavioural Scale	−0.6613	−1.4598, 0.1373	−1.6231	.1046	23.7939	<.001
Difficult temperament*	3	Lab‐TAB, Carey Infant Temperament Scale	−0.3144	−0.5966, −0.0322	−2.1834	.0290	6.567	.0369

Note

If the *Q* statistic was significant (*P *< .05), the random effects size model was used to compute the pooled effect size. If the *Q* statistic was not significant (*P *> .05), the fixed effects size model was used to compute the pooled effect size.

* Significant *P *< .05.

### 3.4 Negative affect

Negative affect is determined by establishing level of sadness, fear, soothability and activity level^28^ and is linked to the infant's ability to regulate their emotional state. Four studies were included in the analysis of negative affect. A total of 16 394 infants (12 043 not exposed and 4351 exposed) between 48 hours and 9 months old were assessed on one of four measures: NBAS, Lab‐TAB, Carey Infant Temperament Scale and Infant Behaviour Questionnaire‐Revised. Individual study effect sizes ranged between −0.806^29^ and −0.02.^7^ Due to heterogeneity within the sample (*Q* = 28.222, *P *< .001, *I* ^2^ = 89.37%), the random effects size model is reported. The combined effect size for negative affect is significant (*d *= −0.502; 95% CI = −0.886 to −0.1191; *z *= −2.568, *P *= .010; fail‐safe N = 809). Infants prenatally exposed to smoking showed heightened negative affect.

### 3.5 Attention

Infant attentional abilities are assessed by the degree of energy the infant displays when engaging with the assessment and the level of facilitation required from the examiner to gain the infant's attention.^30^ Five studies were included in the assessment of the attention subcategory, assessing 1251 infants (846 not exposed to nicotine and 405 exposed to nicotine), between 24 hours and 9 months old. Three different assessment scales were used: NBAS, NICU Network Neurobehavioural Scale and NTA. Individual study effect sizes ranged between −1.358^31^ and −0.134,^22^ and there is evidence of heterogeneity within the sample (*Q* = 32.451, *P *< .001, *I* ^2^ = 87.67%). Therefore, the random effects size model is reported. The combined effect size for attention is significant (*d *= −0.635; 95% CI = −1.031 to −0.238; *z *= −3.129, *P *= .001; fail‐safe N = 98). Those exposed to cigarettes showed significantly poorer levels of attention.

### 3.6 Excitability

Excitability measures peak excitement and rapidity of build‐up, which is a reflection of how much stimulation the baby can handle before entering the crying state, indicating higher levels of arousal.^32^, ^33^ A total of 765 infants (625 not exposed and 140 exposed) between 24 hours and 17 days old were included in the four studies analysed for excitability using two different assessment scales (NICU Network Neurobehavioural Scale and the NBAS). Individual study effect sizes ranged between −0.829^32^ and −0.44.^34^ The data are homogeneous (*Q* = 1.873, *P *= .599, *I* ^2^ = 60.13%), and therefore, the fixed effects size model is reported. The combined effect size for excitability is significant (*d *= −0.5697; 95% CI = −0.772 to −0.367; *z *= −5.529, *P *< .001; fail‐safe N = 44). Infants prenatally exposed to cigarettes demonstrated significantly higher levels of excitability.

### 3.7 Irritability

Irritability is assessed by examining the amount of fussing and crying throughout neurobehavioural assessments, again a reflection of their emotional capabilities. Three studies were included in the analysis for irritability with 1316 (552 not exposed and 764 exposed) infants between 56 hours and 3 days old. The NICU Network Neurobehavioural Scale, Graham‐Rosenblith Behavioural Examination and NTA were used. Individual study effect sizes ranged between −1.949^31^ and −0.125.^35^ The random effects size model was used because of heterogeneity within the data (*Q* = 27.185, *P *< .001, *I* ^2^ = 92.64%). The combined effect size for irritability was significant (*d *= −0.600; 95% CI = −1.148 to −0.0519; *z *= −2.145, *P *= .031; fail‐safe N = 29). Infants prenatally exposed to cigarettes were significantly more irritable.

### 3.8 Lethargy

Lethargy measures indicate the energy resources of the infant and are identified by items on the neurobehavioural assessments such as general muscle tone and reaction to the defensive movement by establishing level of movement.^33^ Two studies were included in the analysis for lethargy with 639 infants (583 not exposed and 56 exposed) ranging between 24 hours and 5 weeks in age, tested with the NICU Network Neurobehavioural Scale. Individual study effect sizes ranged from −1.142^10^ to −0.147.^22^ The data are heterogeneous (*Q* = 15.847, *P *< .00, *I* ^2^ = 93.68%); therefore, the random effects size model is reported. The combined effect size for lethargy is not significant (*d *= −0.628; 95% CI = −1.680 to 0.346, *z *= −1.262, *P *= .206). Prenatal exposure to smoking is not significantly related to the lethargy levels of infants tested.

### 3.9 Muscle tone

Muscle tone is identified by assessing how smooth or jerky the infant's movements are and amount of 90° arm movements the infant displays. Additionally, measures such as pulling the infant to sit are used as an indication of muscle tone.^33^ Muscle‐tone weakness is identified in the infant when the majority of movements are jerky, restricted and when there is significant head lag when the infant is pulled to a seated position.^36^ Four studies were included in the analysis for muscle tone with a total of 1101 infants (436 not exposed and 665 exposed), between 56 hours and 5 days old assessed with one of four measures (NICU Network Neurobehavioural Scale, Graham‐Rosenblith Behavioural Examination, NBAS and Neurological Scores). Individual studies had an effect size ranging between −1.010^31^ and −0.308.^35^ The data were homogeneous (*Q* = 6.908, *P *= .074, *I* ^2^ = 56.57%); therefore, the fixed effects size model is reported. The combined effect size is significant (*d *= −0.361; 95% CI = −0.484 to −0.239; *z *= −5.796, *P *< .001; fail‐safe N = 28). Infants prenatally exposed to smoking had significantly more muscle‐tone weakness.

### 3.10 Orientation

Orientation items assess the infant's ability to follow and engage with animate and inanimate objects such as following a face or rattle for example.^33^ A total of 16 556 infants (12 107 not exposed and 4449 exposed) between 48 hours and 9 months old, based on six studies, were included in the subcategory analysis for orientation. The assessments used were the NBAS and Carey Infant Temperament Scale. The range of effect sizes across individual studies was −1.115^31^ and −0.070.^37^ Due to heterogeneity (*Q* = 26.969, *P *= .001, *I* ^2^ = 81.46%) of the sample, the random effects size model is reported. The combined effect size for orientation is significant (*d *= −0.464; 95% CI = −0.757 to −0.171; *z *= −3.104, *P *< .001; fail‐safe N = 98). Infants prenatally exposed to smoking demonstrated significantly worse levels of orientation.

### 3.11 Regulation

Regulation is assessed by the infant's abilities to self‐sooth, for example whether they need support in settling down following a period of crying,^33^ emphasising their emotional self‐soothing abilities. A total of 16 597 infants (12 238 not exposed and 4359 exposed), between 48 hours and 9 months old, were analysed in the subcategory for regulation, based on six studies using three different assessment measures (NICU Network Neurobehavioural Scale, NBAS and Carey Infant Temperament Scale). Individual study effect sizes ranged between −0.782^38^ and −0.067.^22^ This was a heterogeneous sample (*Q* = 11.250, *P *= .046, *I* ^2^ = 55.55%), and therefore, the random effects size model is reported. The combined effect size for orientation abilities was significant (*d *= −0.261 (95% CI = −0.4411 to −0.082; *z *= −2.864, *P *= .004; fail‐safe N = 82). Infants prenatally exposed to smoking showed significantly more problems in their ability to regulate their behaviour.

### 3.12 Stress

Infant stress is a reflection of the autonomic nervous system and as such is determined by whether colour changes in the face or body occur, number of startles and whether tremors can be seen throughout the assessment.^33^ A total of 695 infants (612 not exposed and 83 exposed), between 24 hours and 5 weeks old, were tested using a single assessment measure, the NICU Network Neurobehavioural Scale, across three studies. Individual study effect sizes varied between −1.510^32^ and −0.002.^22^ Due to heterogeneity in the sample (*Q* = 23.793, *P *< .001, *I* ^2^ = 91.59%), the random effects size model was used. The combined effect size for stress was not significant (*d *= −0.661; 95% CI = −1.459 to 0.137; *z *= −1.623, *P *= .104). Infants prenatally exposed to smoking did not show significantly higher stress compared with non‐exposed infants.

### 3.13 Difficult temperament

Difficult temperament of the infant, that is fussiness, irritability and negative affect throughout the assessment, is used to determine the infant's temperament.^29^ A total of 192 infants (116 not exposed and 73 exposed) between 56 and 6 months old were assessed in three studies using the Lab‐TAB and the Carey Infant Temperament Scale for temperament. Individual studies reported effect sizes between −0.556^39^ and −0.134.^37^ Because of the heterogeneity within the sample (*Q* = 6.596, *P *= .036, *I* ^2^ = 69.68%), the random effects size model was used. The combined effect size for temperament was significant (*d *= −0.314; 95% CI = −0.596 to −0.032; *z *= −2.183, *P *= .029; fail‐safe N = 14). Infants prenatally exposed to cigarette smoke demonstrated higher levels of difficult temperament in comparison with infants not prenatally exposed to smoke.

## 4 DISCUSSION

The aim of this systematic review and meta‐analysis was to establish which areas of neurobehaviour are most strongly related to prenatal cigarette exposure in infants up to one year of age. Overall, the results support the claim that prenatal exposure to smoking is associated with a range of neurobehavioural consequences in infants within the first year of life. Eight of the 10 subcategories that were analysed in the meta‐analysis indicate that prenatal smoking is significantly associated with poorer neurobehavioural functioning in infancy. Measures of negative affect, attention, excitability, irritability and orientation demonstrated medium significant effects, with regulation, difficult temperament and muscle‐tone weakness, indicating smaller significant effects. Stress and lethargy tests, however, did not result in any significant pooled effects.

We argue that the neurobehavioural deficits evident in infants of mothers who smoke cigarettes reflect early behavioural dysregulation associated with prenatal exposure to cigarette smoking. The metabolites of cigarette smoke, carbon monoxide and nicotine, interfere with the normal placental functioning acting as a vasoconstrictor, with uterine blood flow being restricted to roughly 38%.^4^, ^40^, ^41^ Carbon monoxide is likely to lead to foetal hypoxia depriving the developing brain of oxygen and nutrients required for typical brain development. Such effects can be seen in prenatally exposed newborns whose cerebral oxygen saturation level is lower in comparison with infants not exposed.^42^ This interpretation is supported by studies using animal models.^43^, ^44^ Similarly, studies highlight the widespread effects of nicotine affecting a range of neurotransmitters, brain regions and systems which disrupt brain development. Specifically, the neurotransmitter nicotine acetylcholine plays a role in supporting the development of infant regulatory behaviours, such as temperament.^35^, ^43^ Differences in neurobehaviour of infants prenatally exposed to cigarettes are based on changes in brain functioning as a result of carbon monoxide and nicotine exposure.^4^

Research indicates that mother‐infant relationships are under more stress, that is less responsiveness and emotional interactions, if the infant displays neurobehavioural deficits in areas such as affect, with infants demonstrating reduced eye contact and/or reduced smiling during parent‐infant interaction.^45^ This type of unresponsiveness by the infant leads to a negative feedback loop during mother‐infant interactions. As this review indicates, maternal smoking during pregnancy is related to deficits in infant neurobehavioural functioning; for example, infants prenatally exposed to cigarettes are likely to be more irritable compared to non‐exposed infants. A more irritable child will affect quality of parenting behaviours which have negative effects on the infant including less stimulation, less responsiveness and less physical contact.^46^ Because of these negative parenting engagements, the infant's neurobehavioural development is further dysregulated due to reduced interactions.^31^ As a result, an infant who lacks stimulation and physical contact is more likely to show delays in their motor development.^47^ This delay in turn will be an additional strain on the already stressed mother‐infant relationship. Long‐term attentional and behavioural problems can be reflective of these early deficits in neurobehavioural functioning of an infant.^48^

### 4.1 Limitations

The relationship between neurobehavioural developmental factors and prenatal cigarette smoke exposure is complex, often associated with a number of covariates such as preterm birth, gestational age at birth, maternal demographics and substance use (eg alcohol).^23^, ^24^ As shown in Table 2, these types of variables were controlled for in the effect size analysis in the majority of studies. Nevertheless, other covariates such as maternal psychological factors were not considered in many of the studies reviewed, despite the known effects on infant neurobehaviour. For example, maternal antenatal stress and anxiety are positively related to infant outcomes including behavioural and cognitive development such as regulation difficulties, irritability and poorer attention.^49^ Given that these factors were not controlled for in all the studies analysing the effect of cigarette exposure, it was difficult to determine in our current review the extent to which these factors may have influenced the test results.

Due to such confounding variables, it is possible that studies claiming to find a relationship between prenatal smoke exposure and subsequent infant neurobehaviour are measuring an indirect relationship rather than a true causal effect.^50^, ^51^ As a consequence of the epidemiological nature of this research, not all potential confounds can be controlled for and it is difficult to carry out a true experimental design as cigarette exposure cannot be randomly assigned, thus highlighting a methodological limitation.^52^ However, by synthesising the available evidence across multiple populations and study designs, this meta‐analysis strengthens the case for a true causal effect between cigarette exposure and infant neurobehaviour.^50^, ^51^

It is notable however that by studying infants up to one year of age (the range of ages of infants studied is shown in Table 2), we cannot rule out the possibility that in the older infants the effects of their mothers' smoking on neurobehavioural outcomes were due to postnatal rather than prenatal exposure.^9^ Furthermore, the amount of cigarette exposure and at what time point exposure occurred (including postnatal exposure) differed between studies. In the early stage of development, there is naturally a lot of variation and disorganisation in the neurobehavioural profile of infants since the brain is not fully developed at birth,^53^ and environmental factors influence brain development.^54^ Therefore, we have to consider whether the differences seen in infant neurobehavioural development are short‐term or long‐term factors and whether the negative consequences can be reduced or potentially eliminated through neurobehavioural interventions.

## 5 CONCLUSIONS

The results from the meta‐analysis indicate that exposure to prenatal cigarette smoking is associated with negative neurobehavioural outcomes in infants up to one year of age. Research indicates that not all women believe that smoking has negative behavioural consequences for their infant.^55^ Thus, examining neurobehavioural differences in smoke‐exposed and non‐exposed foetuses and infants is essential in order to convince pregnant women to abstain from cigarette consumption during their pregnancy and after birth. For example, smoking during pregnancy may result in irritable infants which cry more than infants with a calm temperament.^37^ The current review and analysis provides further support of the negative effects prenatal smoke exposure has on infant neurobehaviour within the first year of life.

## CONFLICT OF INTEREST

None.
