# Supplementary material for: Infant neurobehavioural consequences of prenatal cigarette exposure: A systematic review and meta‐analysis
Source: Acta Paediatr. 2020 Jan 24;109(6):1112–24. doi: 10.1111/apa.15132 (PMC7317476; doi:10.1111/apa.15132)
Supplement: Supplementary file 1 [file APA-109-1112-s001.docx]

**Table S1.** ROBINS-I: tool for risk of bias.

| Study | Bias due to confounding | Bias in selection of participants into the study | Bias in classification of interventions | Bias due to deviations from intended interventions | Bias due to missing data | Bias in measurement of outcome | Bias in selection of the reported result | Overall bias |
| --- | --- | --- | --- | --- | --- | --- | --- | --- |
| Barros et al., (2011) ^(10)^ | Low | Low | Low | Low | Low | Low | Low | Low |
| Espy et al., (2011) ^(56)^ | Low | Low | Low | Low | Low | Low | Low | Low |
| Godding et al., (2004) ^(57)^ | Low | Low | Low | Low | Low | Low | Low | Low |
| Hernandez-Martinez et al., (2012) ^(15)^ | Low | Low | Low | Low | Low | Low | Low | Low |
| King et al., (2017) ^(58)^ | Low | Low | Low | Low | Moderate | Low | Low | Moderate |
| Law et al., (2003) ^(32)^ | Low | Low | Low | Low | Low | Low | Low | Low |
| Mansi et al., (2007) ^(31)^ | Low | Low | Low | Low | Low | Low | Low | Low |
| Mundy (2009)a ^(39)^ | High | Low | Low | Low | Low | Low | Low | Moderate |
| Mundy (2009)b ^(39)^ | High | Low | Low | Low | Low | Low | Low | Moderate |
| Pickett et al., (2008) ^(37)^ | High | Low | Low | Low | Low | Low | Low | Moderate |
| Saxton (1978) ^(38)^ | High | Low | Low | Low | Low | Low | Low | Moderate |
